# Fc–FcγRI Complexes: Molecular Dynamics Simulations Shed Light on Ectodomain D3′s Potential Role in IgG Binding

**DOI:** 10.1021/acsomega.4c06318

**Published:** 2024-11-28

**Authors:** Aslı Kutlu, Eda Çapkın, Kaan Adacan, Meral Yüce

**Affiliations:** † Istinye University, Faculty of Natural Science and Engineering, Department of Molecular Biology and Genetics, 34396 Istanbul, Türkiye; ‡ Sabanci University, Faculty of Engineering and Natural Sciences, 34956 Istanbul, Türkiye; § Sabanci University, SUNUM Nanotechnology Research and Application Center, 34956 Istanbul, Türkiye; ∥ Imperial College London, Department of Bioengineering, SW7 2AZ London, United Kingdom

## Abstract

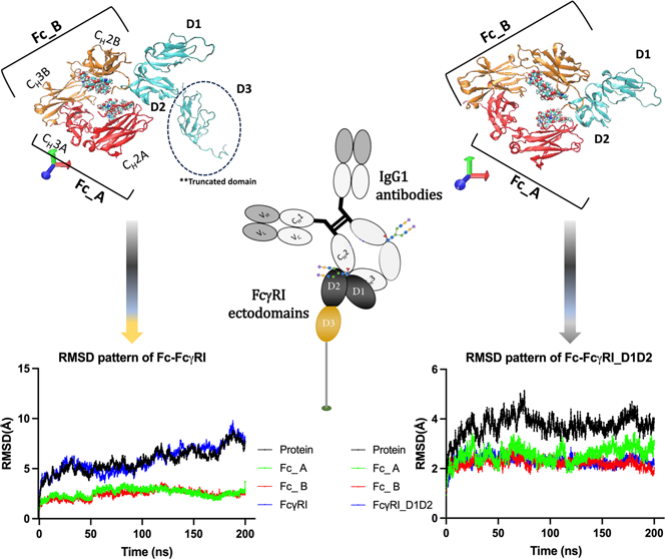

FcγRI plays a crucial role in the effector function of IgG antibodies, interacting with the lower hinge region of IgG1 with nanomolar affinity. Binding occurs specifically in domain 2 (D2) of the FcγRI ectodomain, while domain 3 (D3) is a flexible linker. The D3 domain is positioned away from the IgG binding site on the FcγRI and does not directly contact the Fc region. This study investigates the structural and functional properties of FcγRI D3 using 200 ns classical MD simulations of two models: (1) a full FcγRI ectodomain complex with Fc and (2) a truncated model excluding D3. Our findings suggest that the D3 ectodomain provides additional structural flexibility to the FcγRI–Fc complex without altering the C backbone motion or flexibility of the KHR binding motif in the FG loop. Critical residues involved in binding and contributing to complex stability were evaluated regarding changes in intramolecular interactions and destabilization tendency upon D3 truncation. Truncation did not significantly alter interactions around glycan-interacting residues in Fc chains or FcγRI–Fc binding interfaces. These findings provide valuable insights into the role of FcγRI D3 in modulating the structural dynamics of the FcγRI–Fc complex. While D3 does not directly contact Fc, its mobility and positioning may modulate the receptor’s affinity, accessibility, and ability to bind IgG immune complexes. We suggest that a truncated FcγRI construct lacking the D3 domain may be a promising candidate for biosensor or capturing agents’ development and optimization, offering improved performance in IgG capture assays without compromising critical binding interactions.

## 1 Introduction

Fc γ receptor RI (FcγRI) has the highest affinity for IgG1-type monoclonal antibodies within the Fc γ receptor family (FcγRs). The surface of immune cells such as monocytes, macrophages, dendritic cells, and neutrophils expresses it.^1^ The structure of FcγRI consists of an ectodomain where interactions are formed through the lower hinge region of the Fc part of IgG1, transmembrane, and intracellular domain. FcγRI ectodomain has three domains called D1, D2, and D3. Among these ectodomains, the IgG1 interaction specifically occurs through D1–D2 domains. FcγRI is triggered by the engagement of the antibody on the target cell and leads to antibody-dependent cellular cytotoxicity (ADCC), complement-dependent cytotoxicity (CDC), or antibody-dependent cellular phagocytosis (ADCP). The immune response could be altered depending on the glycosylation profile of the monoclonal antibody.^2−5^

The structural analyses suggest that the D3 domain within the ectodomain region in FcγRI provides a higher affinity than FcγRIIa and FcγRIIIa, consisting of only D1 and D2 ectodomains. Besides, the KHR motif in the D2 domain provides a positive charge on the FcγRI and stabilizes the interaction between the FcγRI and Fc chains. The glycans within the Fc chains contribute to binding interactions forming H-bonds or van der Waals (VDW) interactions, but they are not directly involved in binding events.^2,6,7^ Experimental and computational studies further investigate the effects of different glycans on the binding affinity. The binding interactions with IgGs vary depending on the types of glycans, bringing the allosteric effect of antigen binding for the FcγRI ectodomain.^8−11^

The D3 domain in the FcγRI ectodomain is reported as a linker with no direct role in the binding interaction with IgG. The first characterization study for FcγRI proposed that the D3 domain was positioned away from the binding site and put the hinge region of IgG in a straight conformation toward the cell surface. According to the literature, molecular dynamics (MD) studies performed with full FcγRI ectodomains reported that the D3 domain provides flexibility for the overall protein structure.^8^ Still, there is a lack of information about D3′s structural and functional role in the FcγRI–Fc complex. Also, several variants were introduced into the FcγRI ectodomain to investigate their impacts on the binding affinity for IgG1, and the presence of truncated variants in the D3 domain reported a slightly decreased affinity compared to native.^1,12^

Previously, our group reported the FcγRI (CD64) as a ligand molecule for site-specific IgG1 capture and showed its potential in biosensor applications for tumor necrosis factor-α detection.^10,11^ In this study, we aimed to understand more about the structural and functional roles of the D3 domain in the Fc–FcγRI complex by performing 200 ns classical MD simulations with and without the D3 domain. For this purpose, the protein complex and its domains were evaluated for backbone structure (RMSD), flexibility (RMSF), intramolecular interactions, and change in stabilization tendency. The preservation of critical interactions within Fc and FcγRI–Fc complexes was also evaluated in the presence and truncation of the D3 ectodomain. These calculations have revealed that the truncated version of FcγRI could also be offered for better IgG capturing from complex samples in biosensor applications due to its restricted mobility without altering the critical interactions existing in Fc and Fc–FcγRI complexes.

## 2 Methodology

### 2.1 System Preparation and Simulation

For all MD studies, a 4X4M Protein Data Bank (PDB) file was used for this study. The protein and glycan systems were prepared via the CHARMM GUI input generator,^13−17^ using the glycan reader module. Also, the mutated residues within the PDB file (4X4M) were modified to the native FcγRI ectodomain (UNIPROT: P12314) structure to mimic biological conditions by CHARMM GUI. The solvation of the entire system was performed by adding transferable intramolecular potential 3P (TIP3P) water models, and then the system was neutralized by adding 0.15 mM NaCl. Two different models were constructed: (1) full model of Fc: FcγRI and (2) truncated Fc: FcγRI model by excluding the D3 ectodomain. Within the entire model, 6X His-Tag at its C-terminus is used to facilitate the easy recovery of a protein complex from cell harvest, and it is included to mimic the biological condition exactly. The full and truncated Fc: FcγRI models comprised 415,222 and 269,381 atoms, respectively, including water and ions. The NAMD program was conducted with CHARMM 36 all-atom force fields to perform MD simulations. Electrostatic interactions were calculated using the particle mesh Ewald (PME) method.^18,19^ To control the pressure at 1 atm, the Langevin dynamic was used for NpT ensembles. Before production simulations, the system was minimized in 10,000 steps via the Greedy Algorithm, followed by equilibration of the entire system for 1 ns at 298 K as NpT. For all simulations, the periodic boundary conditions were applied in all dimensions (*x*, *y*, *z*). Along with 200 ns, the production simulations were collected as an NpT ensemble with a 2 fs/step integration velocity at 310 K. All equilibration and production simulations were independently repeated 3 times to avoid artifacts.

### 2.2 Analysis of Molecular Dynamics Trajectory

MD trajectory data was analyzed in terms of backbone root-mean-square deviations (RMSDs), average Cα root-mean-square fluctuations (RMSFs), salt bridge interactions, and dihedral angle calculations (phi and psi angles) via visual molecular dynamics (VMD)^20,21^ In addition, the calculations of single-residue RMSD-RMSF were performed with the vmdICE 1.0 plugin in VMD. The single-residue RMSD and RMSF calculations represent the change in RMSD/RMSF values of the selected residue for each time point along the entire MD trajectory to provide time-dependent information about the dynamics of the selected residue. FoldX, which was implemented as a YASARA plug-in with saved protein conformations every 10 ns along the whole MD trajectory, calculated the changes in the destabilization tendencies of truncated and complete models of Fc, FcγRI, and Fc–FcγRI complexes.^22−24^ HawkDock tool is utilized to perform MM/GBSA calculations of full and truncated Fc–FcγRI complexes (10.1093/nar/gkz397). The saved conformations in every 10 and 200 ns MD trajectories are used to perform HawkDock. For both full and truncated models of Fc–FcγRI complexes, Fc chains and FcγRI ectodomains were designed as receptor and ligand, respectively, to perform docking in which HawkDock scores are calculated. Among the top 10 models generated by HawkDock, the best conformation is selected to run MM/GBSA calculations to end up with free-energy decomposition analysis. Δ*G* (kcal/mol) Graphics were plotted by GraphPad Prism (v.10) as an average of all three independent simulations. FoldX and MM/GBSA results included the standard deviations of the plots for each data point.

## 3 Results and Discussion

### 3.1 RMSD and RMSF Studies

**Figure 1:**
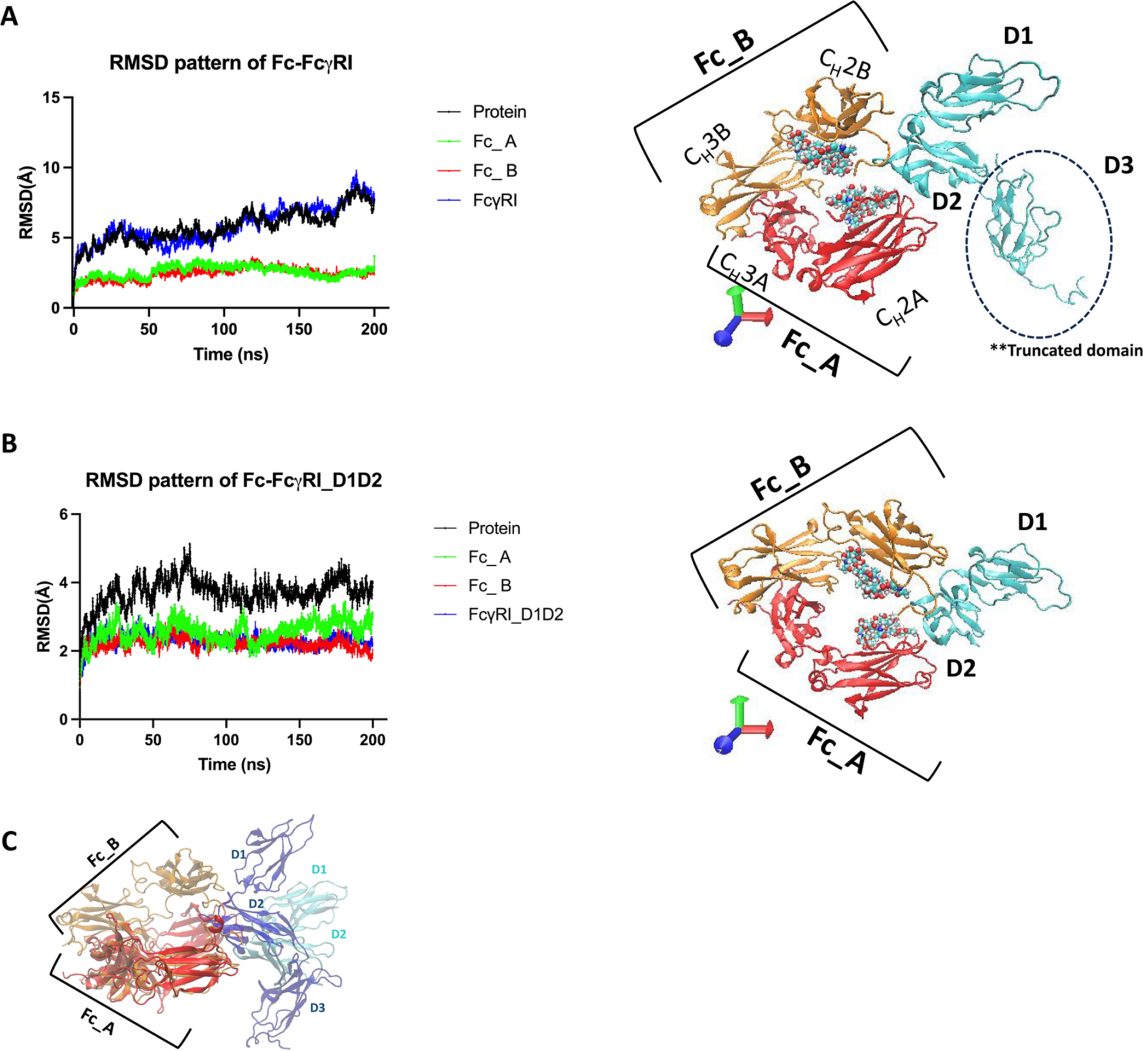
Evaluation of RMSD values of the full and truncated FcγRI: Fc complexes over 200 ns cMD. (A) RMSD plot for the protein (FcγRI: Fc), Fc chains, and the FcγRI ectodomain within the full model. (B) RMSD plot for the protein (FcγRI: Fc), Fc chains, and FcγRI ectodomain within the truncated model. FcγRI ectodomain is shown in cyan. The Fc region is shown as FcA (red) and FcB (orange) chains. The glycans were positioned at Asn297 of FcA (red) and FcB (orange). The protein domains are drawn in New Cartoon formats, and the glycans are drawn in the VDW format using visual molecular dynamics (VMD) tools. (C) The superpositions of the last frames (200th ns) of FcγRI: Fc_full and FcγRI: Fc_truncated complexes come from the Repeat #1 system. All protein domains are drawn in a New Cartoon format using visual molecular dynamics (VMD) tools. FcA and FcB chains of FcγRI: Fc_full and FcγRI: Fc_truncated systems are colored orange/edgyglass and red/opaque, respectively. FcγRI parts of full and truncated systems are drawn in blue/edgyglass and cyan/opaque, respectively.

We began to present our calculations, starting with RMSD calculations over a trajectory of over 200 ns MD. As presented in Figure 1A, RMSD patterns of Fc–FcγRI and FcγRI were similar to each other (in ∼5.8 Å range), and RMSD patterns of FcA and FcB chains were reported with ∼2.6 and ∼2.45 Å, respectively, in the case of including whole ectodomains (D1–D3). Upon the truncation of the D3 domain in FcγRI, there was a significant decrease in the backbone motion of Fc–FcγRI and FcγRI with ∼3.7 and ∼2.3 Å, respectively, and there was a little decrease in RMSD patterns of FcA and FcB as ∼2.5 and ∼2.2 Å (Figure 1B). There were 40 and 50% reductions in the backbone motion of Fc–FcγRI and FcγRI complexes with the truncation of the D3 domain, and this implied the critical role of the D3 domain in their mobility. It was interesting to notice that the backbone motions of Fc chains varied by displaying higher backbone motion in FcA than FcB by referring to the asymmetrical binding of FcγRI, as suggested by Kiyoshi et al.^1^ (see Figure S1).

Specifically for Fc chains, the truncated model has resulted in higher RMSD values compared to that of the full model, except C_H_3A, by implying that the loss of interactions occurs between the D2 domain of FcγRI and Fc chains. In detail, the average RMSD values were decreased from 2.1 Å in C_H_2A to 2.2 Å in C_H_2A_truncated and from 2.1 Å in C_H_2B to 1.9 Å in C_H_2B_truncated (Figure S1). Also, in CH3B, the average RMSD values decreased from 1.3 to 1.5 Å upon the truncation of the D3 domain (see Figure S1). The interesting point is that more drastic alterations were reported in the backbone motion of C_H_2B and C_H_3B domains compared to those of C_H_2A and C_H_3A, even though C_H_2B and C_H_3B domains were located at the most distal proximity to the D3 domain in FcγRI. More details about Fc chains and RMSF calculations of full and truncated models were provided. According to RMSF patterns, higher flexibility has been reported for C_H_2B and C_H_3B domains in the full model compared to those in truncated domains (Figure S2A,B). Full model C_H_2A and C_H_2B RMSF values were found to be 1.5-fold and 1.2-fold higher than those of the truncated model, respectively. For C_H_3A and C_H_3B, the changes in RMSF values were 1.6 and 1.8 higher than that of the truncated model, respectively. This difference in RMSD and RMSF results of Fc chains refers to their asymmetric binding to FcγRI receptors that are utilized further to alter the effector response of therapeutic monoclonal antibodies.^25^

**Figure 2:**
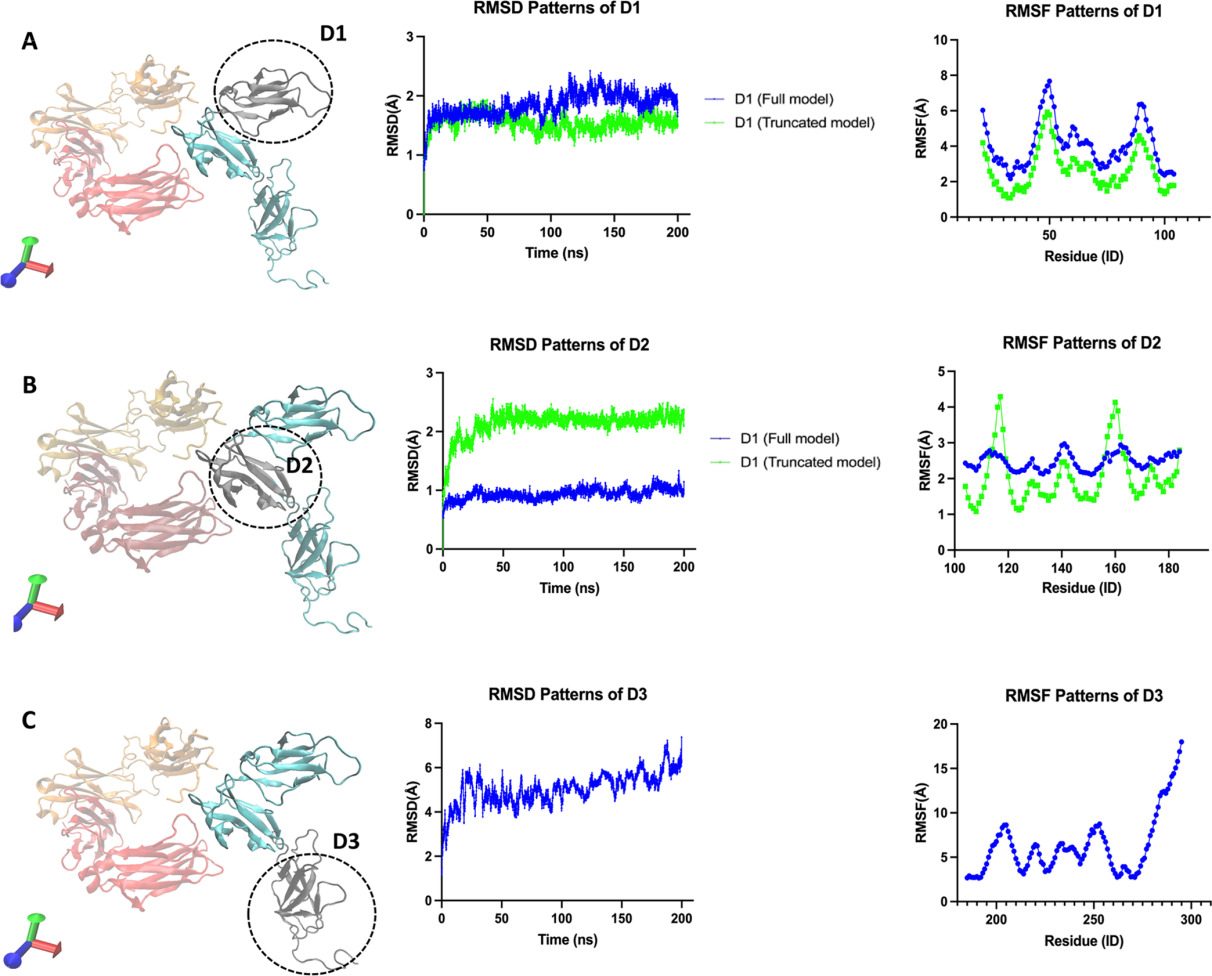
Evaluation of FcγRI ectodomain for RMSD and RMSF patterns over 200 ns: (A) RMSD and RMSF values of the FcγRI ectodomain D1 on the full and truncated models, (B) RMSD and RMSF values of the FcγRI ectodomain D2 on the full and truncated models, and (C) RMSD and RMSF values of the FcγRI ectodomain D3 on the full and truncated models. The Fc region is shown as FcA (red) and FcB (orange) chains. FcγRI ectodomain (D1–D3) is shown in cyan/gray and drawn in a New Cartoon format using visual molecular dynamics (VMD) tools.

Then, the next thing was to understand how the reorganization of FcγRI ectodomains was affected in the case of the D3 domain absence. In Figure 2, RMSD patterns of each ectodomain (D1–D3) were presented by confirming the role of the D3 domain in providing molecular mobility (Figure 2C). Among the D1 and D2 ectodomains, the D2 domain was the most affected by the truncation of D3 such that there was a higher mobility in the D2 domain with average RMSD values of 0.9 Å in full and in 2.1 Å truncated model (Figure 2B). Specifically, there was no crucial change in the backbone motion of D1 in the case of either the presence or absence of the D3 domain in the FcγRI (see Figure 2A). Our findings correlate with the literature study in which the FcγRI ectodomain (PDB ID: 3RDJ) was docked to an Fc chain of IgG1-type antibody, and a noticeable change was observed with the D3 domain.^1^ Also, Asaoka et al.^1,26^ compared the monoclonal antibody binding activity of full FcγRI ectodomain and truncated FcγRI (D1 and D2) to suggest that there is a slight decrease in the binding affinity of truncated FcγRI compared to that of the full model due to the leading decrease in the dissociation constant (*k*_d_) by keeping association constant almost the same. In the literature, the positive correlation between improved mobility of the protein and the accessible binding site(s) of ligand(s) is reported to increase the association rate.^27−29^ Regarding RMSF pattern calculations for FcγRI ectodomains, the most noticeable change was reported only for D2 as being in line with its RMSD pattern. Except for a few residues in D2 (VAL113, PHE114, THR115, GLU116, GLY117, GLU118 and PRO119, and THR158, ASN159, ILE160, SER161, and HIS162), there was a restricted flexibility in D2 domain along 200 ns MD simulation. With the higher RMSF values of these excepted residues (Val113–Pro119 and Thr158–His162), the higher RMSD pattern of the D2 domain in the truncated model has been well explained. The closer structural analysis of VAL113–PRO119 and THR158–HIS162 regions in the D2 domain has suggested that both these regions were in close contact with D3 by creating a binding interface between D2 and D3 in FcγRI. Upon the truncation of the D3 domain, the intramolecular interactions of residues within VAL113–PRO119 and THR158–HIS162 regions have been lost, and thus, the higher residue flexibility has been reported (see Figures 2B and S3A,B). The single RMSF calculations of VAL113–PRO119 and THR158–HIS162 regions in the D2 ectodomain are presented for both Fc–FcγRI_full and Fc–FcγRI_truncated models. Here, it is seen that the truncation of the D3 ectodomain has led to an increase in single RMSF residues for all of these residues. Specifically, we focused on the duration of percentage change in single RMSF values for these selected residues in the truncated model compared to those of the full one along the 200 ns MD trajectory. Here, we set our % change cutoff as 40% in terms of change in single RMSF values coming from Fc–FcγRI_truncated compared to Fc–FcγRI_full one. Here, we report that the percentage of reported higher single RMSF values in the FcγRI: Fc_truncated than our set threshold are 20% in VAL113, 40% in PHE114, 32% in THR115, 51% in GLU116, 49% in GLY117, 46.4% in GLU118, and 26.1% in PRO119. For THR159–HIS162 region, the percentage of reported higher single RMSF values in the FcγRI: Fc_truncated than our set threshold are 50.1% in THR158, 53.6% in ASN159, 59.6% in ILE160, 44.3% in SER161, and 28.6% in HIS162. These results have also supported the fact that there is a loss in the intramolecular interactions involved by residues within VAL113–PRO119 and THR158–HIS162 in the D2 ectodomain upon the truncation of the D3 domain. Hence, it results in the increase in RMSD and RMSF patterns of the D2 ectodomain (see Figure 2B).

### 3.2 Evaluating the Intramolecular Interactions Existing along the Binding Interfaces and Glycans’ Coordination

**Figure 3:**
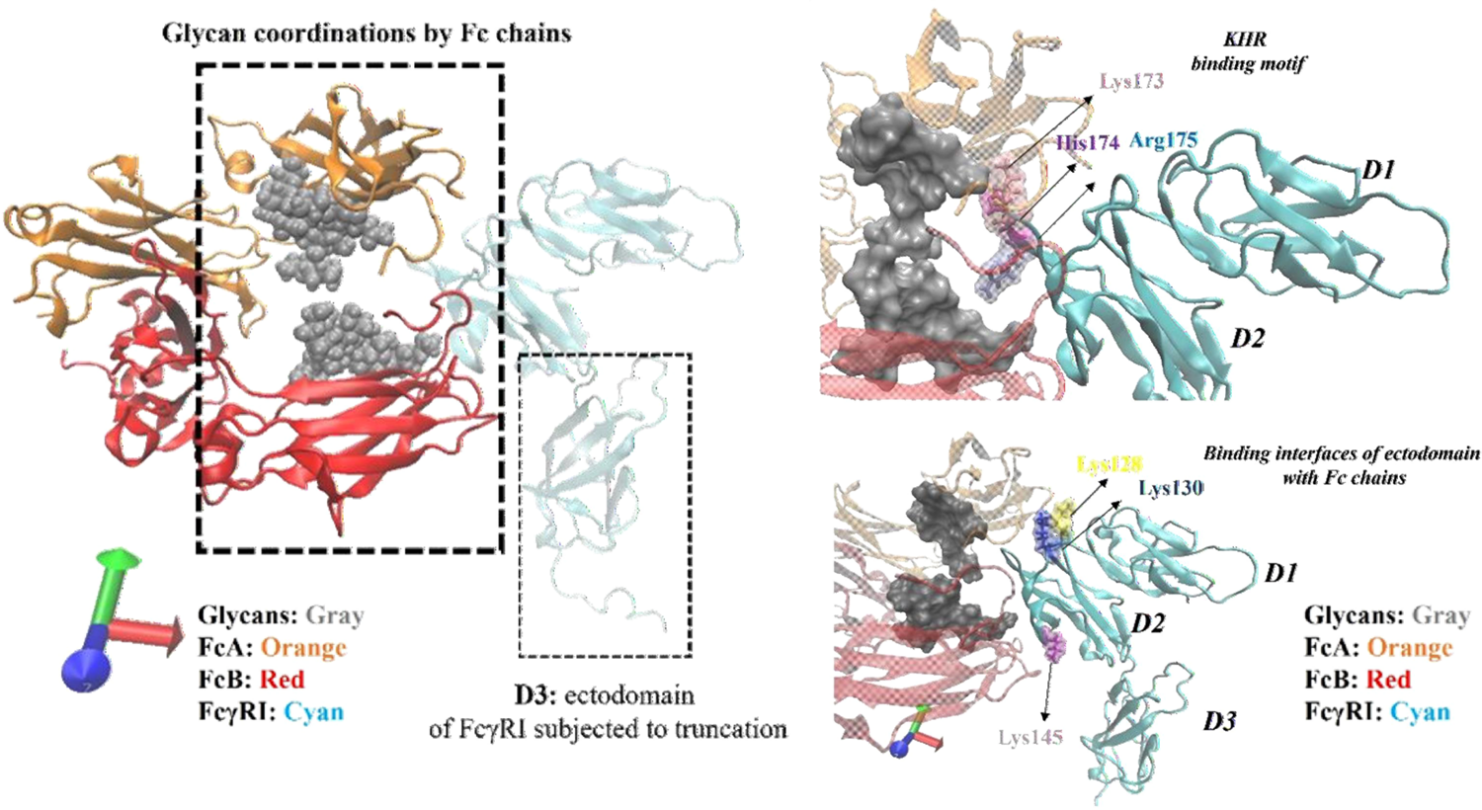
Illustration of binding interactions within the protein complex regarding the critical residues involved in glycan coordination and the binding interfaces between Fc chains and FcγRI. FcγRI ectodomain is shown in cyan, and the Fc region is shown as FcA (red) and FcB (orange) chains. The protein domains are drawn in New Cartoon formats, and the residues are drawn in licorice format using visual molecular dynamics (VMD) tools.

**Figure 4:**
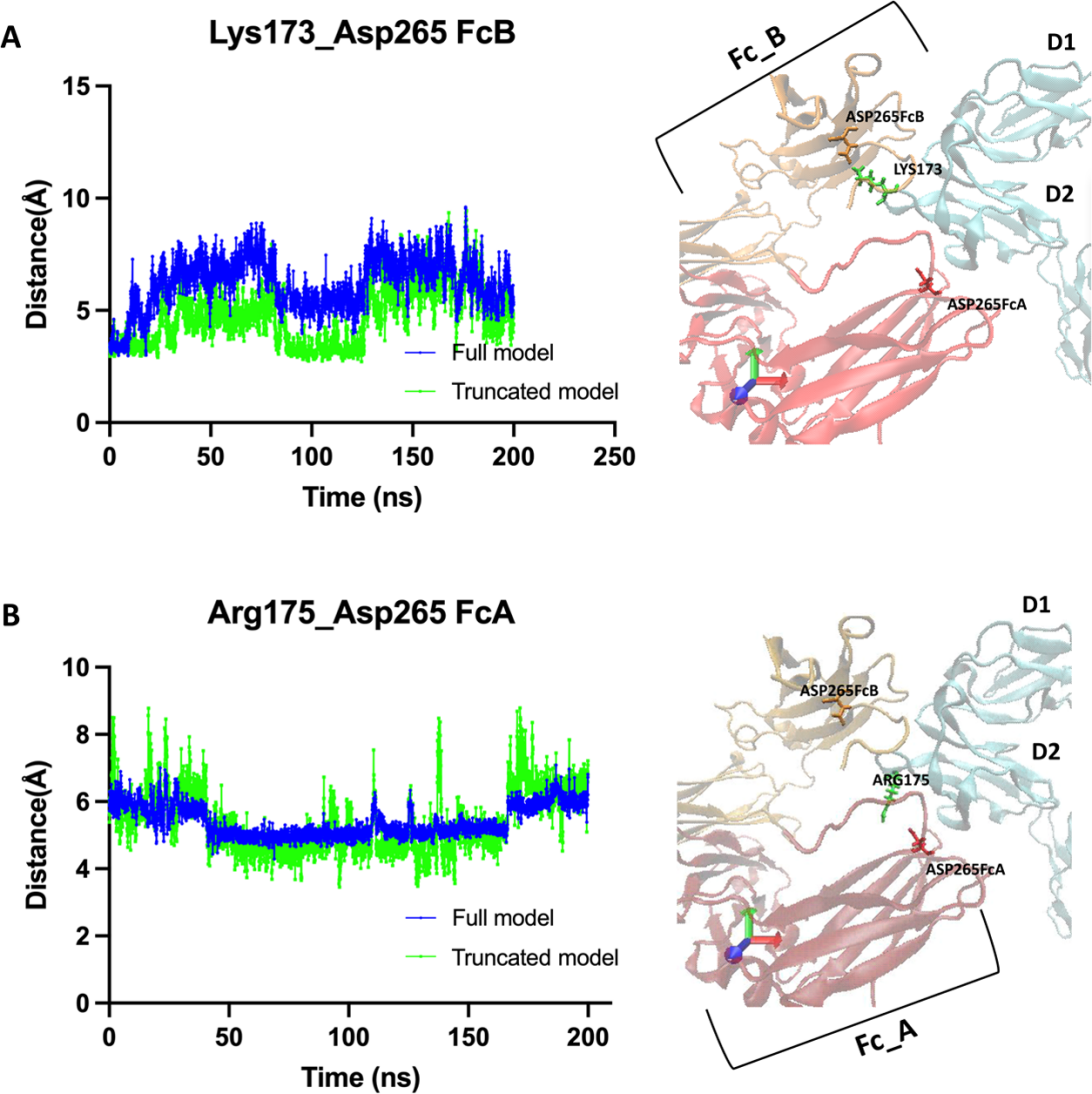
Evaluation of salt bridge interactions on the KHR motif for models. (A) LYS173: ASP265FcB and (B) ARG175: ASP265FcA salt bridge interactions on the whole and truncated models. FcγRI ectodomain is shown in cyan, and the Fc region is shown as FcA (red) and FcB (orange) chains. The protein domains are drawn in New Cartoon formats, and the residues are drawn in licorice format using visual molecular dynamics (VMD) tools.

Along with 200 ns classical MD simulations, the changes in intramolecular interactions are examined within two main perspectives: (1) the critical residues known to interact with glycans and (2) the evaluation of interactions along the binding interfaces. The role of the KHR motif within the FG loop of the FcγRI ectodomain has already been described well as contributing a high affinity toward IgG antibodies and stabilizing the interaction between FcγRI and Fc regions.^1,2,30^ Here, we have evaluated salt bridge interactions around the KHR motif to reveal the D3 domain’s impact on stabilizing the FcγRI–Fc complex (see Figure 3). First, we reported LYS173: ASP265FcB with a stronger and more stable pattern in the truncated model compared to that in the full one (Figure 4A). For ARG175: ASP265FcA salt bridge interactions, there was no change between the full and truncated models; see Figure 4B. Previously, in the literature, the role of the KHR motif on the binding affinity has been demonstrated by experimental studies, which concluded that HIS174 caused a significant reduction in the binding affinity. At the same time, ARG175 had a moderate effect on it. Besides the decrease in the binding interactions, the disrupted glycan interactions with the Fc region of IgG were also reported by emphasizing the role of the KHR motif. Together with these literature findings, our salt bridge calculations have suggested the role of the D3 domain’s truncation in modulating the structural stability of the FcγRI–Fc complex around the KHR motif.^2,30^

Then, we evaluated the changes in intramolecular interactions existing in the binding interface area of the FcγRI ectodomain by focusing LYS128 and LYS130 residues connecting FcγRI in the D2 domain to FcB and the LYS145 residue connecting FcγRI in the D2 domain to FcA chains. The focused salt bridge interactions along this binding interface of the FcγRI–Fc complex were listed such as LYS128:GLU269_FcB, LYS128:ASP270_FcB, LYS130:GLU269_FcB and LYS130:GLU233_FcB, and LYS145:GLU269_FcA. Compared to the KHR motif, the more unstable pattern was reported for truncated models for several salt bridge interactions, e.g., LYS128–ASP270FcB, LYS130–GLU269_FcB, and LYS145–GLU269_FcA (see Figure S4B,C,E), due to the truncation of D3 domain. Along the 200 ns MD trajectory, more or less the exact distances with similar patterns were reported for LYS128:GLU269_FcB and LYS128:ASP270_FcB in both full and truncated models; see Figure S4A,B. It was also interesting to notice that the LYS130:GLU233FcB salt bridge interaction did not exist within both the full and truncated models in our MD trajectory due to reporting higher than 10 Å distances between LYS130 and GLU233(FcB) within most time points along the trajectory; see Figure S4D. Somehow, a similar pattern was also reported for LYS145:GLU269_FcA such that the distances were higher than 5 Å, especially from 150th until the end of the MD trajectory; see Figure S4E.

Along with this binding interface, special attention must be paid to GLU269 (FcA and FcB) and ASP270 (FcA and FcB) involving interactions since these residues are engaged in glycan coordination. From this perspective, there was no critical loss in the salt bridge interactions in which GLU269 and ASP270 served as partners. However, again, the impacts of asymmetric binding between Fc and FcγRI were also observed within the perspective of intramolecular interactions such that the GLU269 interacting partner in the FcA chain was affected less than that in the FcB chain and ASP270 was affected more than GLU269 in terms of loosened interactions within the FcB chain; see Figure S3. Besides the role of ASP270 and GLU269 residues in Fc chains, several residues have already been reported to be involved in glycan interactions, e.g., TYR296, PHE241, PHE243, ARG301, and LYS334. Here, no salt bridge interactions were detected from these listed residues.

Besides salt bridge calculations, we also measured single RMSD and RMSF values with KHR motif residues, residues within the binding interface of the FcγRI–Fc complex, and glycan-interacting residues. As we focused on the KHR motif, we reported increased single RMSD values of LYS173, HIS174, and ARG175 upon the truncation of the D3 domain of FcγRI compared to that of the full model (see Figure S5). This finding has indicated the improved backbone mobility (C_α_) of these residues along our MD trajectory, most probably keeping to existing salt bridge interactions (Figure 4) into proper distance since there was no critical change between the whole and truncated models of the single RMSF calculations of LYS173, HIS174, and ARG175 residues. A similar pattern has also been reported for LYS128, LYS130, and LYS145 residues existing in the FcγRI ectodomain such that there was an increase in their single RMSD values upon the truncation of the D3 domain but displaying all RMSF patterns for both full and truncated models along 200 ns MD calculations; see Figure S6. Among them, the most noticeable change in a single RMSD pattern was recorded for LYS145 upon the truncation of the D3 domain. By referring to the roles of LYS128 and LYS130 residues in FcγRI ectodomain to have interacted with the Fc region of IgGs, we concluded that there were no significant alterations of Fc–FcγRI regarding our salt bridge interaction and single RMSD/RMSF calculations.

Besides these residues, we reported the single RMSD and RMSF patterns of PHE241, ASP270, GLN295, and TYR296, known as glycan-interacting residues in the Fc chains. Of course, we reported varied RMSD patterns of the same residue in FcA and FcB chains for both full and truncated models. Our results have suggested that single RMSD patterns of all of these residues were affected more by FcA truncation than FcB (see Figure S7). Besides these residues, several residues had a role in the indirect coordination of glycan by Fc chains, and they were PHE243, ARG301, and LYS33. According to the literature findings, these residues have contributed to the coordination of glycans by decreasing their conformational flexibility.^2^ Regarding the calculations of single RMSD values of PHE243, ARG301, and LYS334, we reported increased RMSD patterns upon the truncation of the D3 domain (Figure S8) as being in line with what we calculated in single RMSD of PHE241, ASP270, GLN275, and TYR296 residues; see Figure S7. But again, it has been crucial to emphasize that the flexibility pattern of these residues has been reported as similar in both full and truncated models; see Figure S9. All of these single RMSD and RMSF results regarding glycan-interacted residues or ones having a role in glycan interaction in Fc chains have suggested that the truncation of the D3 domain has caused the alteration in the backbone conformation of glycan-interacting residues in Fc chains, but not in their R-groups, and hence the glycans’ coordination have been well preserved in both full and truncated models along 200 ns MD trajectory.

**Figure 5:**
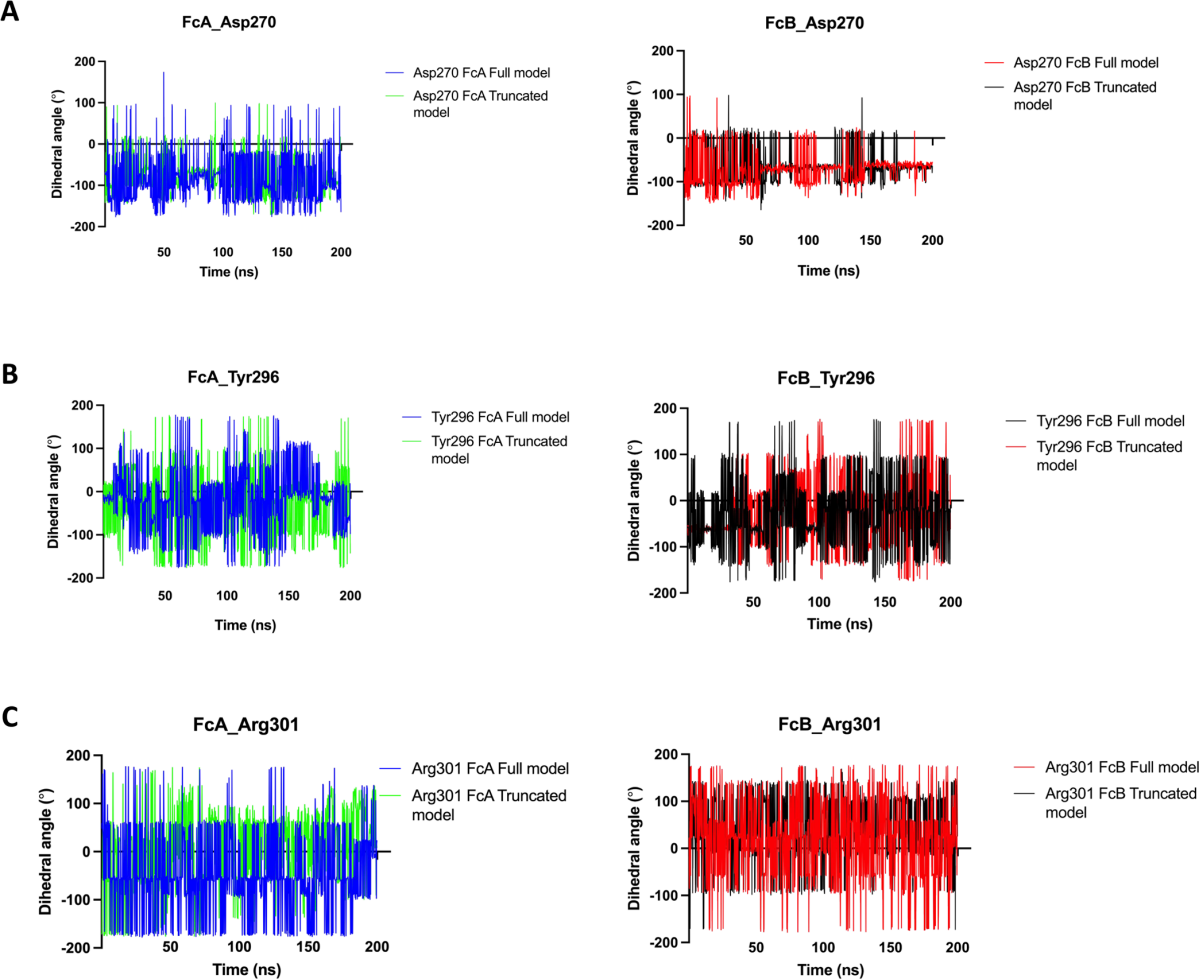
Dihedral phi angle measurements of ASP270 (A), TYR296 (B), and ARG301(C) in FcA and FcB chains along 200 ns MD trajectory.

**Figure 6:**
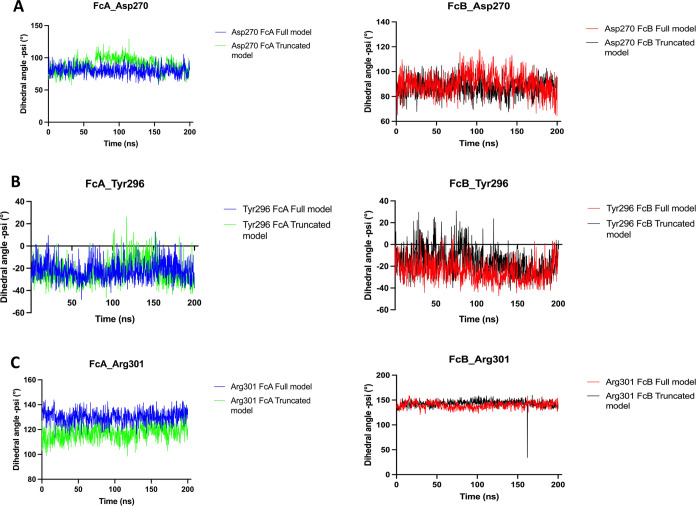
Dihedral psi angle measurements of ASP270 (A), TYR296 (B), and ARG301(C) in FcA and FcB chains along the 200 ns MD trajectory.

In addition to single RMSD and RMSF calculations of glycan-coordinating residues in Fc chains, we also performed the dihedral phi and spsi angle measurements of ASP270 and TYR296 in direct coordination with glycans and ARG301 residue contributing to coordination with glycans in Fc chains due to their trackable change; see Figures 5 and 6, respectively. For all of these residues, asymmetric glycan coordination was observed by comparing full and truncated models for FcA and FcB chains; see Figures 5 and 6. For ASP270 and ARG301 residues, the truncation of the D3 domain in FcγRI did not alter the psi dihedral angle pattern in FcA and FcB chains; see Figure 5A,5C. Specifically for TYR296, we have reported a time lag in its psi dihedral angle pattern upon the truncation of the D3 domain in FcγRI for the FcA chain; see Figure 5B. Still, the conformational integrity within TYR296 has been preserved well, which is critical to coordinate glycans within the Fc–FcγRI complex. Similar to the dihedral phi angle measurements of ASP270, TYR296, and ARG301 residues in FcA and FcB chains, the impacts of asymmetric glycan binding coordinated by ASP270 and TYR296 residues have also been reported in the full and truncated models with the dihedral psi angle calculations; see Figure 6A,B. Specifically for TYR296 in the FcB chain, a little time lag has been reported around ∼80th ns of the whole MD trajectory upon truncating the D3 ectodomain in the Fc–FcγRI complex. The overall structural integrity has been well preserved for all of these critical residues by referring to the dihedral psi angle calculations; see Figure 6.

### 3.3 Revealing the Change in Conformational Stability upon the Truncation of D3 Ectodomain

**Figure 7:**
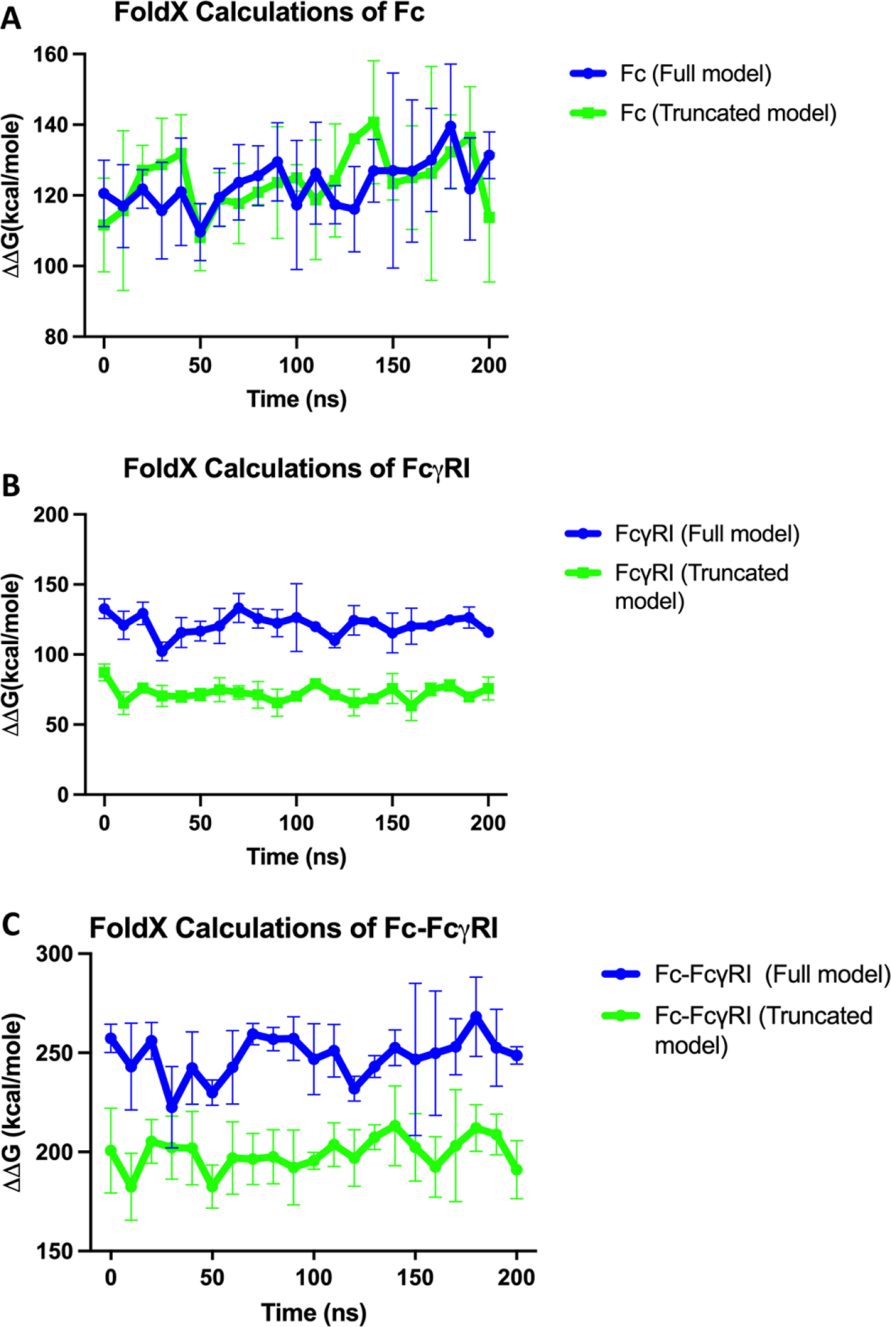
Change in the destabilization tendency of the full and truncated models of Fc chains (A), FcγRI (B), and the Fc–FcγRI complexes (C).

As the last part of the calculations, the impacts of D3 truncation on the stability of Fc, FcγRI, and Fc–FcγRI complexes were evaluated via FoldX in which the saved protein conformations every 10 ns were used. The changes in the stability of Fc, FcγRI, and Fc–FcγRI complexes were calculated as ΔΔ*G* (kcal/mol) values presenting the differences between the Δ*G* of folded and unfolded states of macromolecules. Among FoldX calculations of Fc, FcγRI, and Fc–FcγRI, the more significant impact of D3 truncation was reported in the FcγRI along our MD trajectory. In Figure 7B,C, it is seen that the truncation of D3 ectodomain has resulted in the formation of more stable FcγRI and Fc–FcγRI complexes by referring to the limitations on molecular mobility of these complexes, and that was also confirmed by RMSD/RMSF calculations (Figures 1 and 2). It was interesting to notice that the truncation of D3 ectodomain has also brought an impact on the structural stability of Fc chains even being distantly located (see Figure 7A), and its impact on the stability of Fc chains was different from those of FcγRI and Fc–FcγRI.

Between the time scales of 20–40th and 120–140th ns, the truncation of the D3 domain has increased the destabilization tendency of Fc chains. Still, according to free-energy calculations conducted by Kralj et al., this destabilization referred to by ΔΔ*G* was minimized by 150th ns until the end of the MD trajectory.^27^ With MD simulations of Fc and full-length antibodies, FcγRIIa and FcγRIIIa contributed to interactions with the Fab region of the IgGs, resulting in enhanced binding activity for IgGs. The CH1 domain within the Fab region of the antibody was primarily responsible for binding interactions by the formation of many H-bonds. An MD study for FcγRI evaluated four types of structures, which were free antibody, free FcγRI, antibody: FcγRI complex, and antigen: antibody: FcγRI complex. They indicated that the conformation of the Fc region of the antibody altered to facilitate the FcγRI interactions upon the antigen binding.^7^ Thus, our calculations have suggested that the structural integrity of the Fc–FcγRI complex has been preserved well, even with the truncation of the D3 ectodomain, which allows the Fc region to facilitate FcγRI interactions upon antigen binding.

Besides the FoldX calculations ending up with ΔΔ*G* (kcal/mol) values for Fc–FcγRI_full and Fc–FcγRI_truncated models, we have also performed docking analysis by using the HawkDock tool in which Fc chains and FcγRI ectodomain were set as receptor and ligand, respectively. Regarding HawkDock outputs, we report no reasonable hit for the receptor and ligand couples with few Fc–FcγRI conformations coming from different repeat MD trajectories, e.g., 100th ns conformation of Fc–FcγRI_truncated coming from repeat #1, 40th ns conformation of Fc–FcγRI_full coming from repeat #2, etc. Before reporting docking scores and corresponding MM/GBSA scores for the docked conformations of the Fc–FcγRI_full and Fc–FcγRI_truncated models, we performed normality analysis with docking scores and MM/GBSA scores. Regarding our statistical analysis, it has been concluded that all data sets have passed the Anderson–Darling test for normality test for docking and MM/GBSA calculations, except the repeat #1 data set for docking analysis with 1.206 A^2^ value and repeat #3 data set for MM/GBSA calculation with 1.666 A^2^ value (see Figure S10A,B). As displayed in Figure S10C, there are no critical differences in docking scores of Fc–FcRI systems upon the truncation of the D3 ectodomain. Also, for MM/GBSA calculations, the truncation of the D3 ectodomain has resulted in better Δ*G* (kcal/mol) values compared to that of the Fc–FcγRI_model along 200 ns MD trajectory, except 180th ns conformation. These calculations have also supported the FoldX results, such that the truncation of the D3 ectodomain in FcRI has resulted in structurally well-integrated protein conformation.

## 4 Conclusions

The classical MD simulations employed in this study characterized the dynamic properties of the D3 ectodomain in the FcγRI structure and its relation to structure–function. Two systems were generated: a full-length FcγRI complexed with the Fc region of IgG and a truncated version lacking the D3 ectodomain. The simulations revealed that the D3 ectodomain contributes to higher mobility and flexibility in the D1 and D2 domains of FcγRI, which are associated with lower structural stability. Consistent with the previous literature, our RMSD and RMSF calculations of the FcA and FcB chains within the protein complex confirmed the asymmetry of the Fc region.

Truncation of the D3 ectodomain altered the backbone properties of the Fc chains and the D1 and D2 domains in the FcγRI structure. Notably, the significant glycan–Fc interactions and interactions along the Fc–FcγRI interfaces were well preserved upon the D3 ectodomain truncation. FoldX calculations further confirmed the improved structural stability of the Fc–FcγRI complex in the truncated model.

Based on these structural analyses, the truncated FcγRI protein is suggested to be advantageous for IgG-capturing approaches in sensing and purification applications due to its enhanced structural stability following D3 ectodomain truncation. Additionally, this truncated construct may offer advantages in the recombinant production of FcγRI ectodomains, facilitating easier recovery and higher protein yields from crude samples.

In conclusion, our findings indicate that a truncated FcγRI construct lacking the D3 domain could be a promising candidate for IgG capture in biosensor applications. The reduced flexibility of the truncated FcγRI–Fc complex may lead to more stable and reproducible binding interactions, making it an attractive target for further optimization and development in analytical applications. While the truncated FcγRI construct shows promise based on its improved structural properties, more research is needed to fully assess its potential as an affinity ligand for IgG purification. Challenges around binding affinity, specificity, scalability, and optimization would need to be addressed before it could be widely adopted for industrial-scale IgG purification.

## Data Availability

All data, including replicates, are available in the GitHub repository at https://github.com/meralyucekurt/FcGammaRI.git

## Supporting Information Available

RMSD plot evaluation of the Fc chains over 200 ns, specifically the CH2 and CH3 domains within the Fc structure for the full and truncated models, is a significant aspect of this study, the Fc region is depicted as FcA (red) and FcB (orange) chains, with the glycans positioned at Asn297 of FcA (red) and FcB (orange), the protein domains are presented in New Cartoon format, and the glycans are shown in Licorice format using visual molecular dynamics (VMD) tools (Figure S1); the RMSF plots for the Fc chain domain in the full and truncated model over 200 ns are significant, these plots provide insights into the dynamics of the Fc chains, with (A) RMSF plots for the CH2 domain within the Fc chains and (B) RMSF plots for the CH3 domain within the Fc chains (Figure S2); single-residue RMSF plots of (A)VAL113–PRO119 and (B) THR158–HIS162 residues in D2 ectodomain of Fc–FcγRI_full and Fc–FcγRI_truncated complexes along 200 ns MD trajectory (Figure S3); the evaluation of the salt bridge interactions for the key residues on the surface of FcγRI ectodomain is a valuable part of this study, this evaluation includes (A) LYS128: GLU269FcB, (B) LYS128: ASP270FcB, (C) LYS130: GLU269FcB, (D) LYS130: GLU233FcB, and (E) LYS145: GLU269FcA salt bridge interactions on the full and truncated model (Figure S4) single-residue RMSD and RMSF plots in key residues within the FcγRI ectodomain on the whole and truncated models (A) LYS173 RMSD and RMSF plots (B) HIS174 RMSD and RMSF plots (C) ARG175 RMSD and RMSF plots, the protein domains are drawn in New Cartoon formats, and the glycans are drawn in VDW format using visual molecular dynamics (VMD) tools, FcγRI ectodomain and Fc chains are gray, and residues (LYS173, HIS174, ARG175) are green (Figure S5); single-residue RMSD and RMSF plots in FcγRI ectodomain on the whole and truncated models (A) RMSD&RMSF plots of LYS128 residue (B) RMSD and RMSF plots of LYS130 residue (C) RMSD and RMSF plots of LYS145 residue, FcγRI ectodomain is shown in cyan, and the residues are drawn in licorice format by visual molecular dynamics (VMD) tools (Figure S6); single-residue RMSD plots in key residues within the Fc chains on the full and truncated models (A) PHE241 (B) ASP270 (C) GLN295 (D) TYR296 RMSD plots (Figure S7); single-residue RMSD plots in critical residues within the Fc chains on the whole and truncated models (A) PHE243 (B) ARG301 (C) LYS334 RMSD plots (Figure S8); single-residue RMSF plots in key residues within the Fc chains on the full and truncated models (A) PHE241 (B) ASP270 (C) GLN295 (D) TYR296 (E) PHE243 (F) ARG301 (G) LYS334 RMSF plots (Figure S9); HawkDock calculations of Fc–Fc γRI complexes of full and truncated models, (A) the Anderson–Darling normality test results of docking scores (B) the Anderson–Darling normality test results of MM/GBSA scores (C) the average docking scores and MM/GBSA scores of full and truncated models (Figure S10) (PDF)
