# Supplementary material for: Fc–FcγRI Complexes: Molecular Dynamics Simulations Shed Light on Ectodomain D3′s Potential Role in IgG Binding
Source: ACS Omega. 2024 Nov 28;9(50):49272–82. doi: 10.1021/acsomega.4c06318 (PMC11656251; doi:10.1021/acsomega.4c06318)
Supplement: Supplementary file 1 — ao4c06318_si_001.pdf [file ao4c06318_si_001.pdf]

Fc-FcγRI Complexes: Molecular Dynamics Simulations Shed Light on Ectodomain D3's Potential Role in IgG Binding

Aslı Kutlu[1,*], Eda Çapkın[2], Kaan Adacan[1], Meral Yüce[3,4,*]

1 Istinye University, Faculty of Natural Science and Engineering, 34396, Istanbul, Türkiye
2 Sabanci University, Faculty of Engineering and Natural Sciences, 34956, Istanbul, Türkiye
3 Sabanci University, SUNUM Nanotechnology Research and Application Center, 34956, Istanbul, Türkiye
4 Imperial College London, Department of Bioengineering, SW7 2AZ, London, United Kingdom
* Corresponding authors

***Corresponding authors:**

asli.kutlu@istinye.edu.tr

m.yuce@imperial.ac.uk

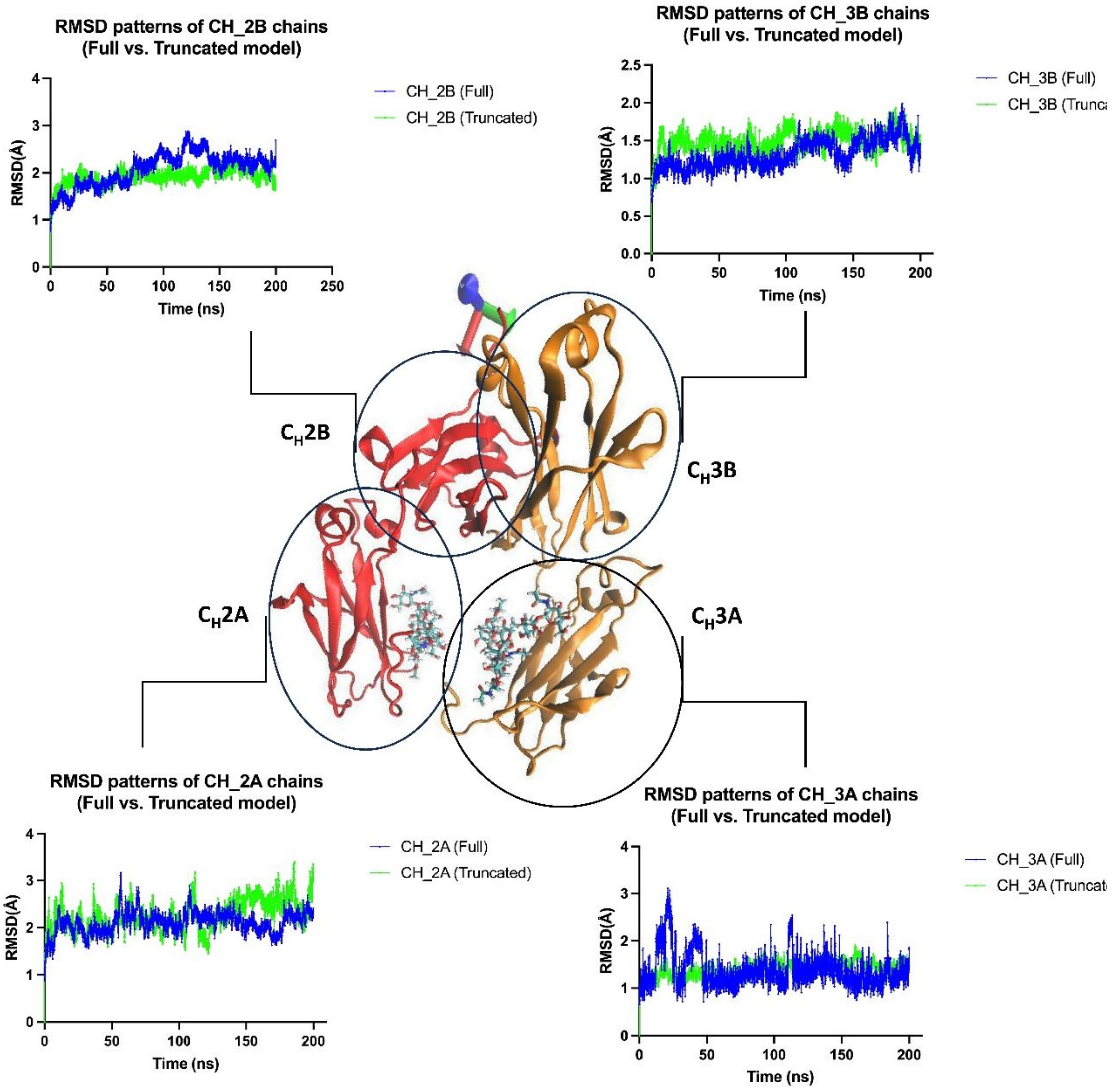

Figure S1. The RMSD plot evaluation of the Fc chains over 200 ns, specifically the CH2 and CH3 domains within the Fc structure for the full and truncated models, is a significant aspect of this study. The Fc region is depicted as FcA (red) and FcB (orange) chains, with the glycans positioned at Asn297 of FcA (red) and FcB (orange). The protein domains are presented in New Cartoon format, and the glycans are shown in Licorice format using Visual Molecular Dynamics (VMD) tools.

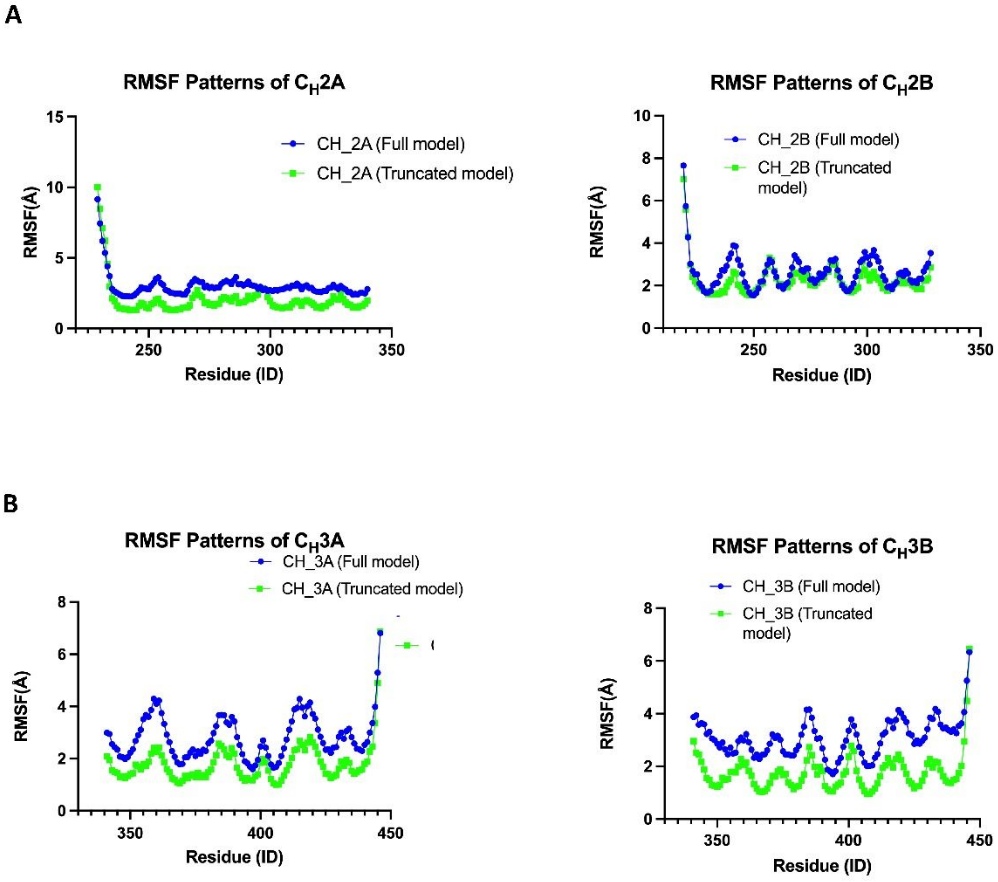

Figure S2. The RMSF plots for the Fc chain domain in the full and truncated model over 200 ns are significant. These plots provide insights into the dynamics of the Fc chains, with A) RMSF plots for the CH2 domain within the Fc chains and B) RMSF plots for the C_H3 domain within the Fc chains.

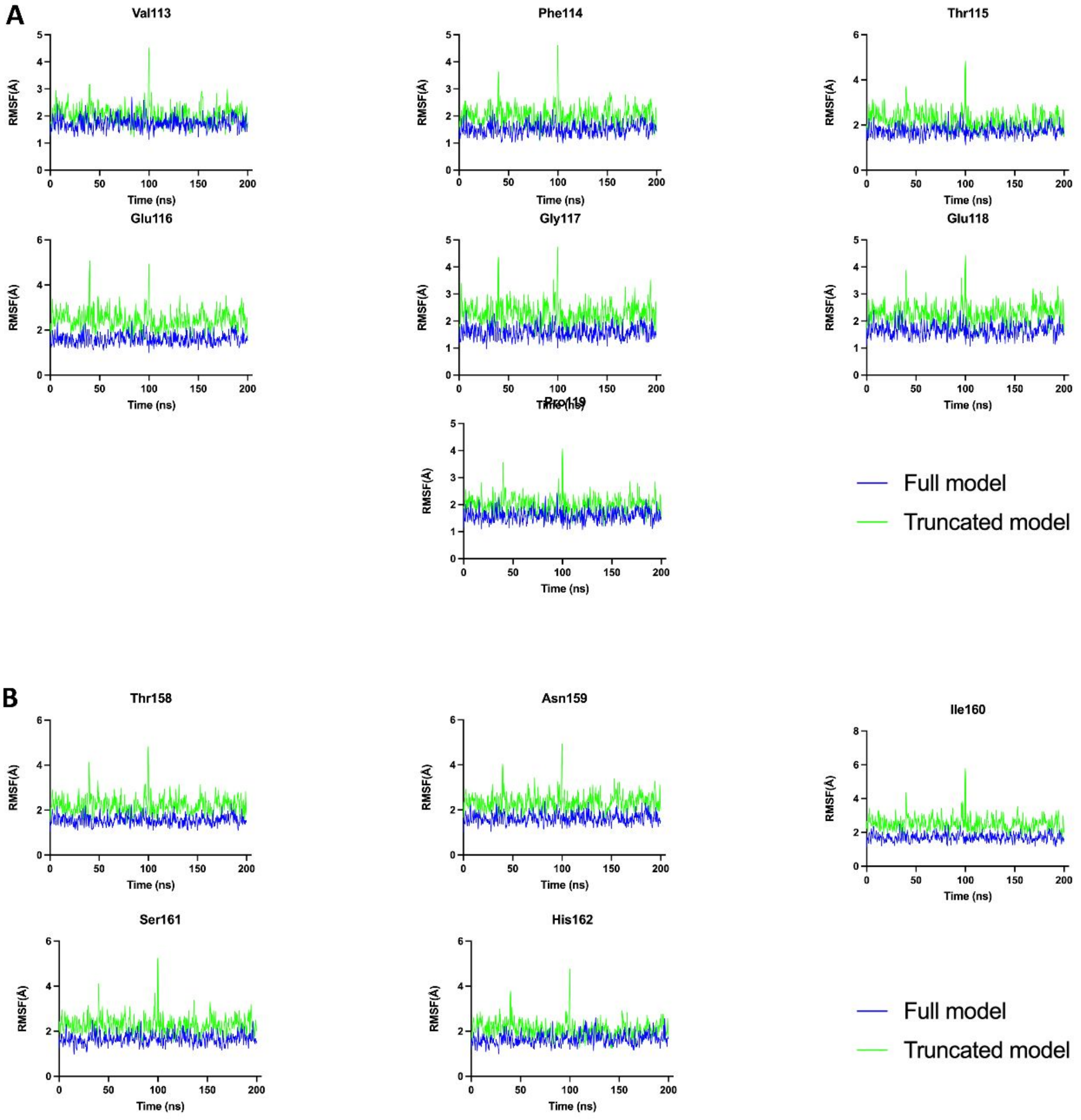

Figure S3. Single residue RMSF plots of (A)VAL113-PRO119 and (B) THR158-HIS162 residues in D2 ectodomain of Fc- FcγRI_full and Fc- FcγRI_truncated complexes along 200 ns MD trajectory.

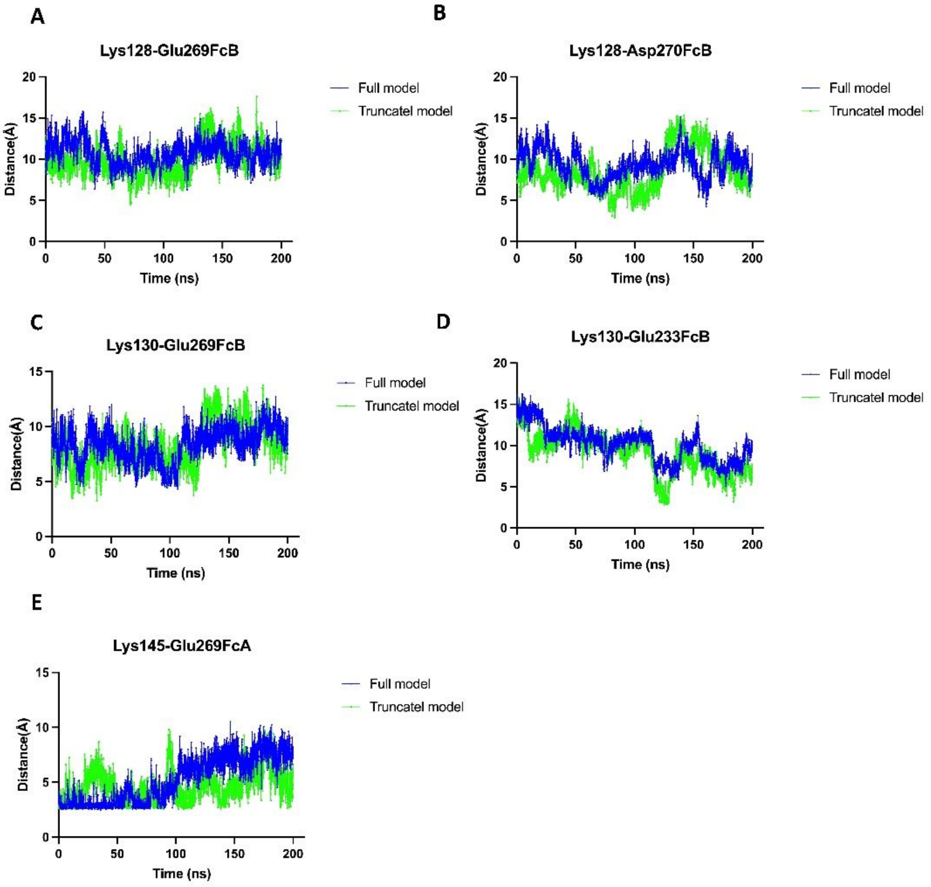

Figure S4. The evaluation of the salt bridge interactions for the key residues on the surface of FcγRI ectodomain is a valuable part of this study. This evaluation includes A) LYS128: GLU269FcB, B) LYS128: ASP270FcB, C) LYS130: GLU269FcB, D) LYS130: GLU233FcB, and E) LYS145: GLU269FcA salt bridge interactions on the full and truncated model

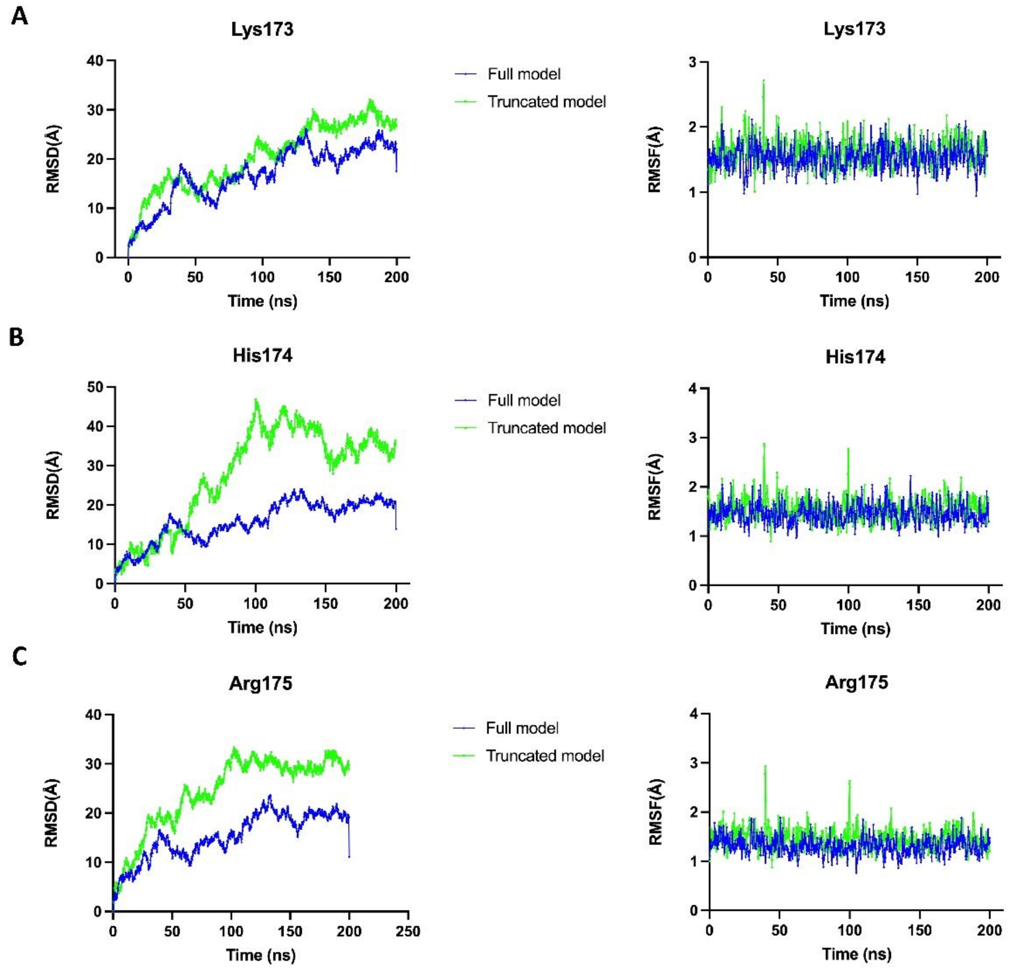

Figure S5. Single residue RMSD and RMSF plots in key residues within the FcγRI ectodomain on the whole and truncated models A) LYS173 RMSD and RMSF plots B) HIS174 RMSD and RMSF plots C) ARG175 RMSD and RMSF plots. The protein domains are drawn in New Cartoon formats, and the glycans are drawn in VDW format using Visual Molecular Dynamics (VMD) tools. FcγRI ectodomain and Fc chains are gray, and residues (LYS173, HIS174, ARG175) are green.

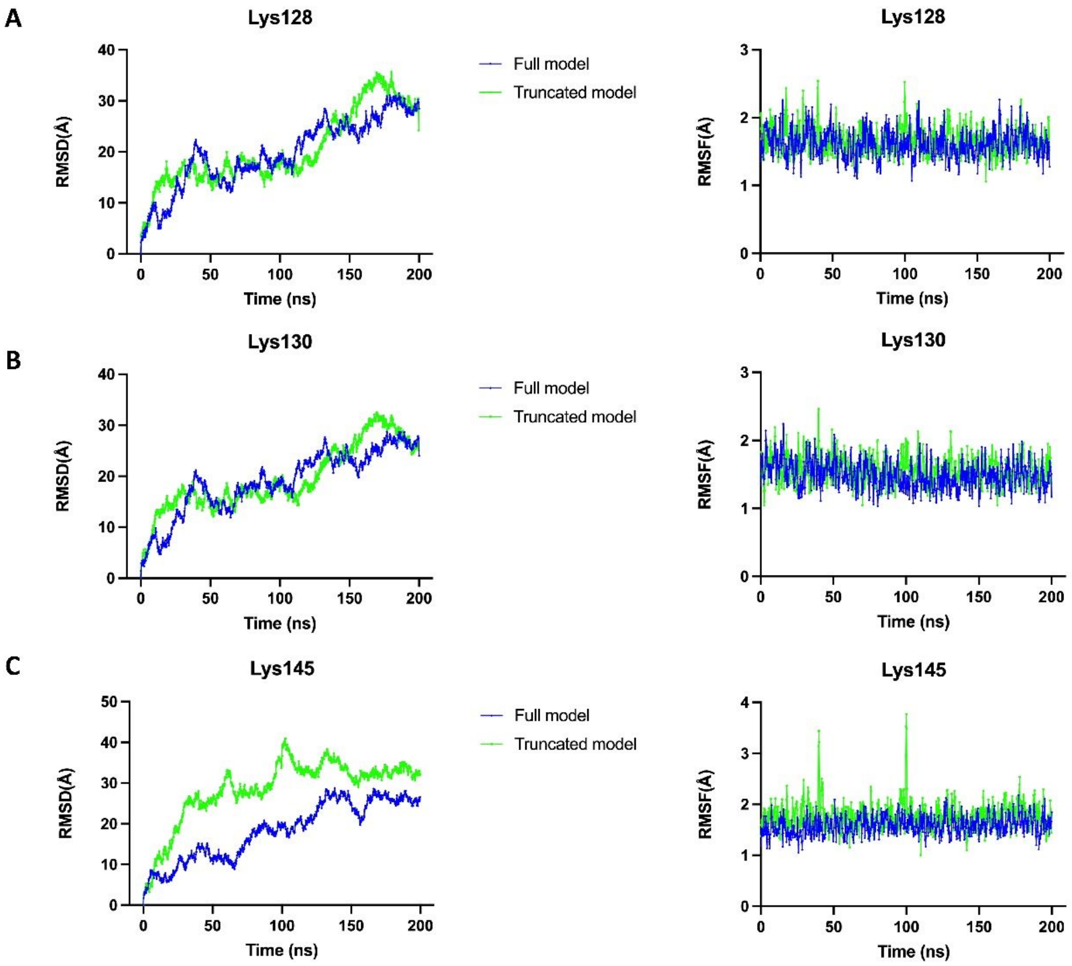

Figure S6. Single residue RMSD and RMSF plots in FcγRI ectodomain on the whole and truncated models A)RMSD &RMSF plots of LYS128 residue B) RMSD and RMSF plots of LYS130 residue C) RMSD and RMSF plots of LYS145residue. FcγRI ectodomain is shown in cyan, and the residues are drawn in licorice format by Visual Molecular Dynamics (VMD) tools.

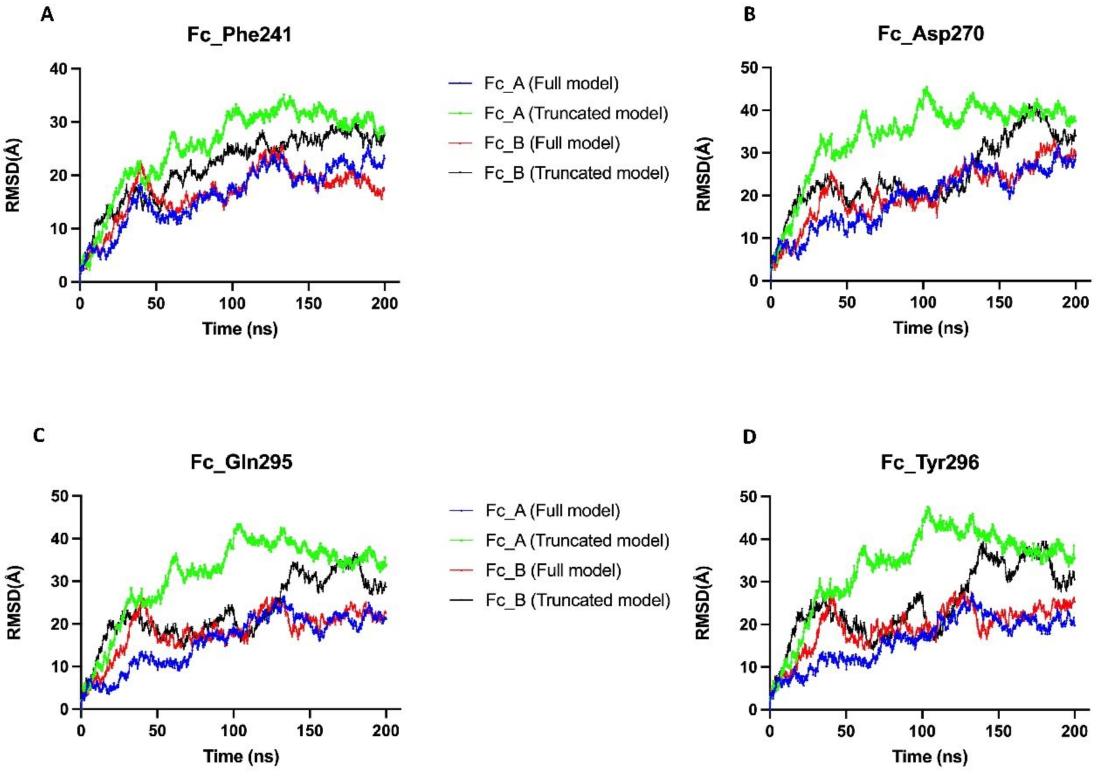

Figure S7. Single residue RMSD plots in key residues within the Fc chains on the full and truncated models A) PHE241 B) ASP270 C) GLN295 D) TYR296 RMSD plots

A

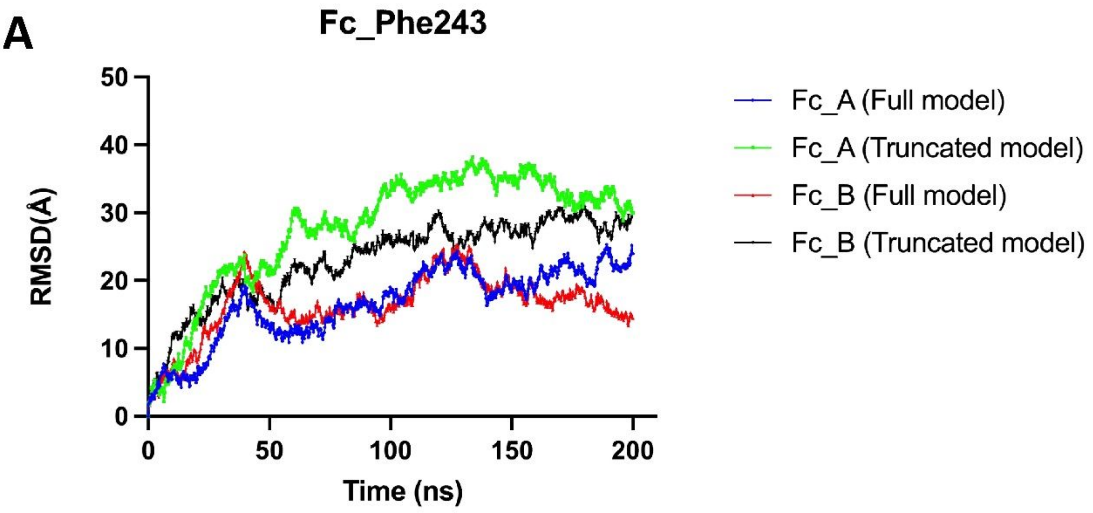

B

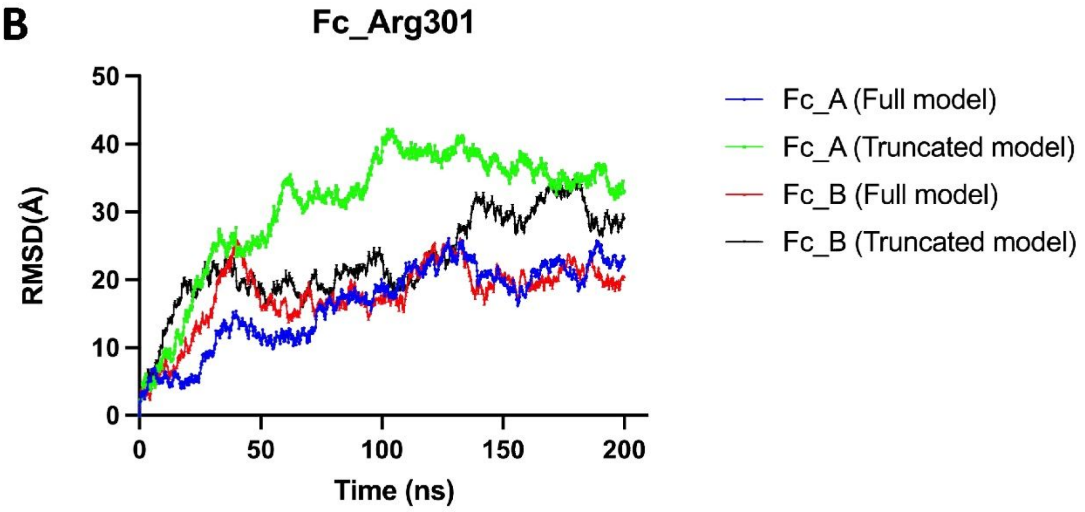

C

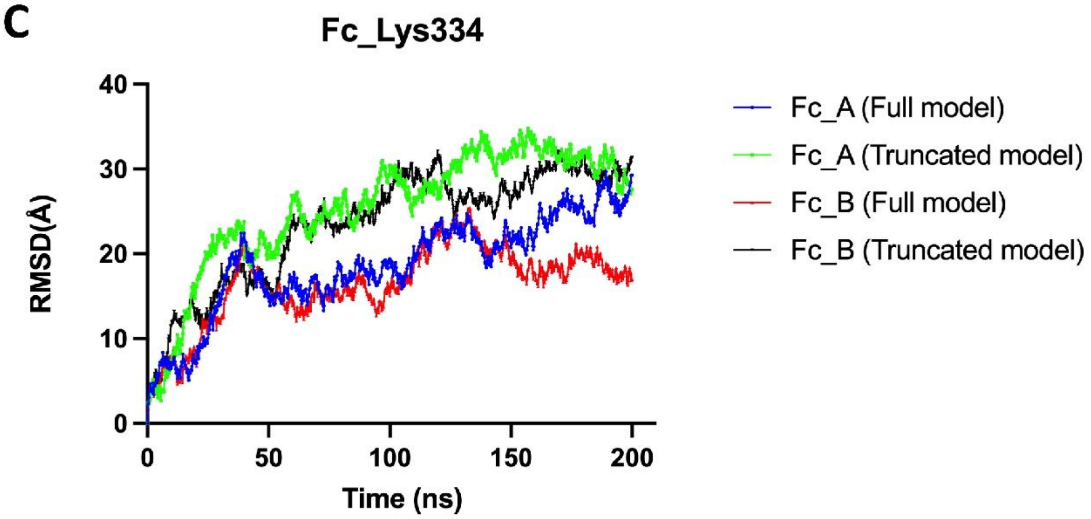

Figure S8. Single residue RMSD plots in critical residues within the Fc chains on the whole and truncated models A) PHE243 B) ARG301 C) LYS334 RMSD plots.

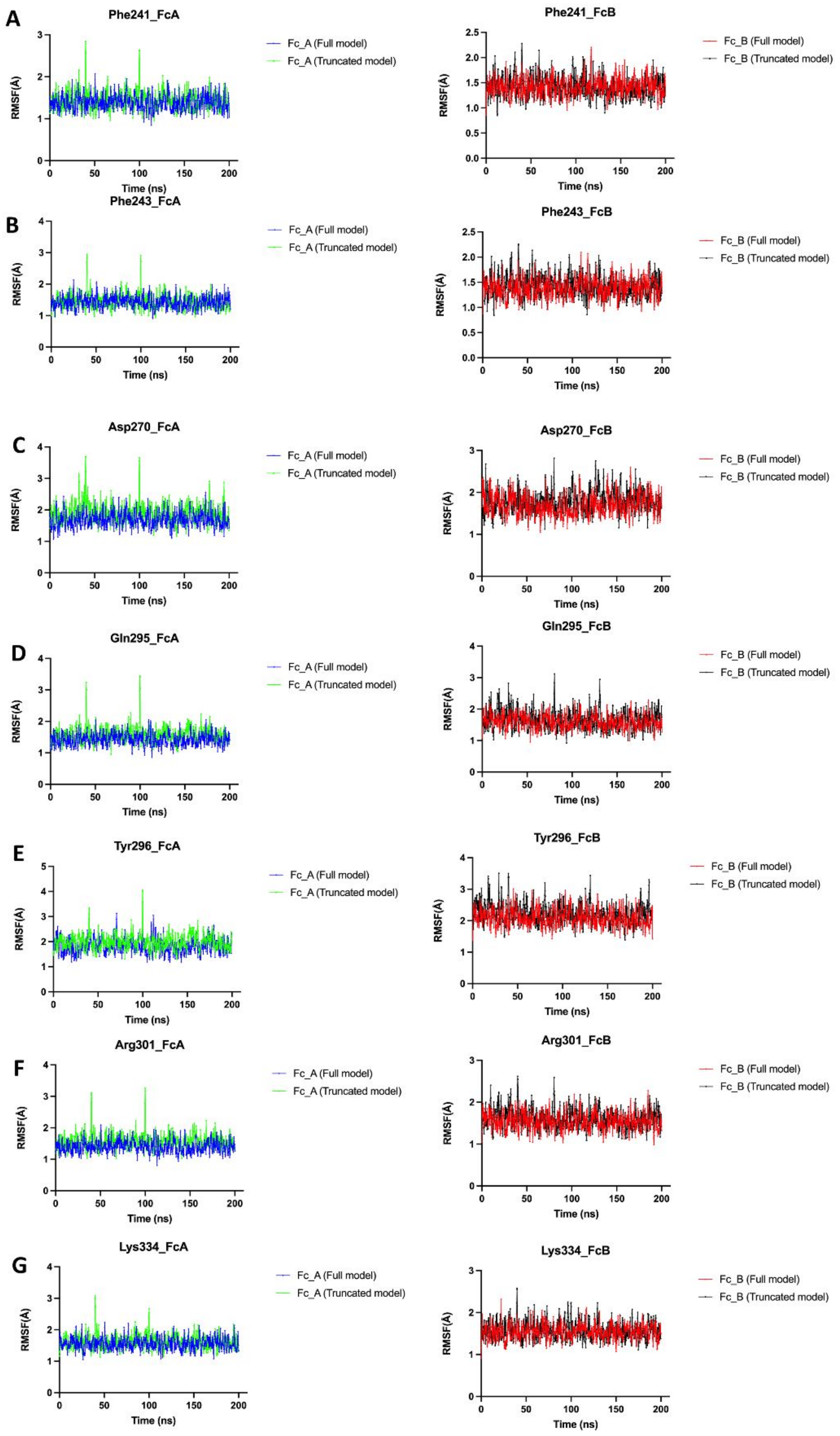

Figure S9. Single residue RMSF plots in key residues within the Fc chains on the full and truncated models A) PHE241 B) ASP270 C) GLN295 D) TYR296 E) PHE243 F) ARG301 G) LYS334 RMSF plots.

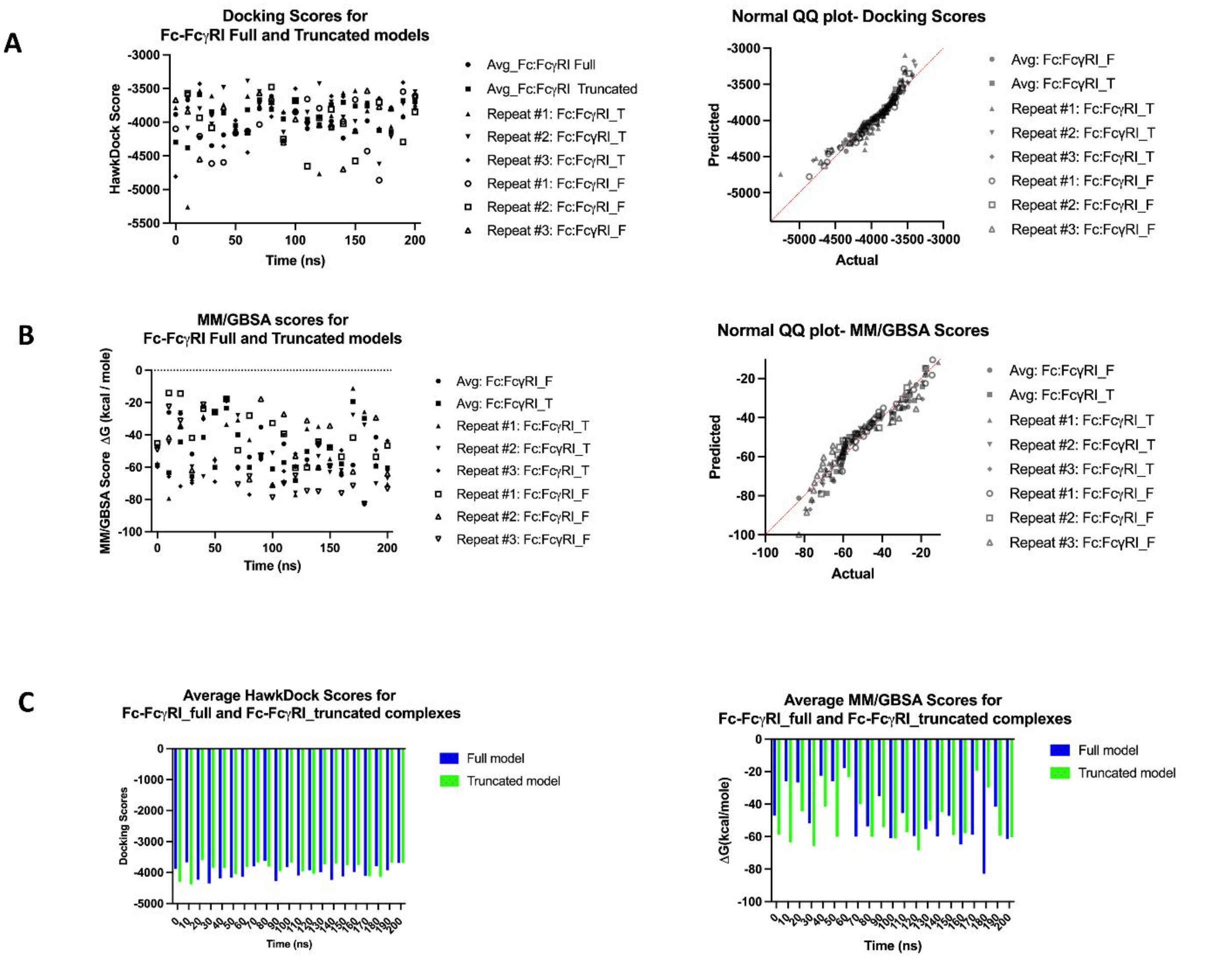

Figure S10. HawkDock calculations of Fc-Fc γRI complexes of full and truncated models. (A) The Anderson-Darling normality test results of docking scores (B) The Anderson-Darling normality test results of MM/GBSA scores (C) The average docking scores and MM/GBSA scores of full and truncated models.
